# Characterization of Liposomes Using Quantitative Phase Microscopy (QPM)

**DOI:** 10.3390/pharmaceutics13050590

**Published:** 2021-04-21

**Authors:** Jennifer Cauzzo, Nikhil Jayakumar, Balpreet Singh Ahluwalia, Azeem Ahmad, Nataša Škalko-Basnet

**Affiliations:** 1 Drug Transport and Delivery Research Group, Department of Pharmacy, Faculty of Health Sciences, University of Tromsø The Arctic University of Norway, N-9037 Tromsø, Norway; jennifer.cauzzo@uit.no; 2 Optical Nanoscopy Research Group, Department of Physics and Technology, Faculty of Science and Technology, University of Tromsø The Arctic University of Norway, N-9037 Tromsø, Norway; nikhil.jayakumar@uit.no (N.J.); ahluwalia@uit.no (B.S.A.); azeem.ahmad@uit.no (A.A.)

**Keywords:** liposomes, nanomedicine, characterization, label-free, quantitative phase microscopy

## Abstract

The rapid development of nanomedicine and drug delivery systems calls for new and effective characterization techniques that can accurately characterize both the properties and the behavior of nanosystems. Standard methods such as dynamic light scattering (DLS) and fluorescent-based assays present challenges in terms of system’s instability, machine sensitivity, and loss of tracking ability, among others. In this study, we explore some of the downsides of batch-mode analyses and fluorescent labeling, while introducing quantitative phase microscopy (QPM) as a label-free complimentary characterization technique. Liposomes were used as a model nanocarrier for their therapeutic relevance and structural versatility. A successful immobilization of liposomes in a non-dried setup allowed for static imaging conditions in an off-axis phase microscope. Image reconstruction was then performed with a phase-shifting algorithm providing high spatial resolution. Our results show the potential of QPM to localize subdiffraction-limited liposomes, estimate their size, and track their integrity over time. Moreover, QPM full-field-of-view images enable the estimation of a single-particle-based size distribution, providing an alternative to the batch mode approach. QPM thus overcomes some of the drawbacks of the conventional methods, serving as a relevant complimentary technique in the characterization of nanosystems.

## 1. Introduction

Nanomedicine emerged as an advanced field expected to change the landscape of pharmaceutical development, promising improved drug efficacy and safety. Various types of nanoformulations (nanocarriers) have been proposed to impart biological superiority [1]. However, many promises remain to be fulfilled, and recent years oversaw the trend of “back-to-basic”, trying to ensure a better understanding of the interplay between drugs, nanocarriers, and biological environment, especially biological barriers [2].

The characterization of a nanosystem is a crucial initial step in the development of novel nanomedicine. Changes in physicochemical properties of a nanocarrier can lead to a change in their behavior, as well as biological fate. Therefore, by tailoring a nanocarrier’s features, we could augment its desired pharmacological effect. However, failure to ensure reliable and robust characterization, within *in vitro* settings, would directly impair the prediction of biological fate and limit success in *in vivo* settings [3].

The carrier size, surface charge, and polydispersity (PdI) are the three major well-established properties known to affect the internalization and potentially the targeting of drug delivery systems within biological environments [4,5,6]. The standard widely utilized characterization techniques are typically batch-mode analyses, such as dynamic light scattering (DLS). Being fast and easy to use, DLS allows the estimation of size distribution and polydispersity index (PdI), which reflects the uniformity of a nanosystem. The combination of DLS and electrophoretic mobility (electrophoretic light scattering) further allows the estimation of the surface charge based on the zeta-potential distribution. Nonetheless, a relevant downside to these techniques is their bias when characterizing polydispersed systems, due to their resolution being limited to a factor of 3, potentially failing to separate multimodal particle distributions [7,8]. Alternative characterization techniques are mostly microscopy-based, namely, transmission electron microscopy (TEM), scanning electron microscopy (SEM), and atomic force microscopy (AFM). These single-particle-size measurement techniques circumvent DLS disadvantage by resorting to a particle-by-particle analysis of the images. However, the widespread use of these techniques is limited by the highly complex sample preparation and their limited accessibility and cost [9].

In addition to physicochemical characterization, it is necessary to assess the behavior of nanosystem in relevant environments. The most common strategy applied to follow the fate of nanosystems is the introduction of a fluorescent label [10]. Fluorescence-based techniques can track nanosystems, potentially both *in vitro* [11] and *in vivo* [12]. Additionally, new methods have been developed to utilize fluorescence in the physicochemical characterization of the nanosystems. Size has been estimated through fluorescent microscopy [13] as well as flow cytometry [14]. Thereof, fluorescent-based techniques are powerful tools to directly establish physicochemical–behavioral relationships. However, the addition of an external component to nanosystems may affect the individual properties of both the nanosystem and the fluorophore [15]. For instance, fluorophores are known to alter nanosystems’ surface properties [16] and to detach from them [11,17]. Furthermore, the fluorescent signal decays with time and is not suitable for long-term tracking. Moreover, all fluorescent techniques reliant on strong illumination can induce high phototoxicity in live biological samples.

New label-free techniques are emerging as a mean to overcome the need for a marker, while attempting to combine physicochemical and behavioral characterizations. Such techniques include surface plasmon resonance (SPR) [18], nanoparticle tracking analysis (NTA) [19], coherent anti-Stokes Raman scattering (CARS) [20], and the technique we utilized in the current work, i.e., quantitative phase microscopy (QPM) [21].

Quantitative phase microscopy (QPM) is a label-free technique that is able to detect nanometer pathlength changes by inducing minimal photo-toxicity to the study sample. QPM setups can be operated in two modes, namely, on-axis and off-axis, depending on the intended application. Off-axis quantitative phase microscopes allow imaging of highly dynamic events. The Fourier transform algorithm is used to reconstruct an image from the interferogram, providing high temporal resolution at the cost of spatial resolution, due to the filtering of object information in the Fourier domain. On the contrary, interferograms from on-axis microscopes can be reconstructed through the phase-shifting algorithm, preserving high-frequency information and high spatial resolution at the cost of temporal resolution, due to their requiring of 4–5 frames per phase per image [22]. The latter setup provides lossless and highly sensitive measurements of the specimens and is thus most suited for the characterization of sub-diffraction limit-sized nanoparticles [23].

Most of the QPM systems are implemented with either highly temporally and spatially coherent light source (laser) or low temporally and spatially coherent light source (white light). These light sources carry certain disadvantages such as speckle noise and coherent noise—when using lasers or chromatic aberration and dispersion—in the case of white light [24,25,26,27,28]. To overcome the challenges associated with conventional light sources, we implemented QPM with spatially low and temporally high coherent light source, also called pseudothermal light source (PTLS). Details for such type of light source can be found elsewhere [29,30].

**Figure 1:**
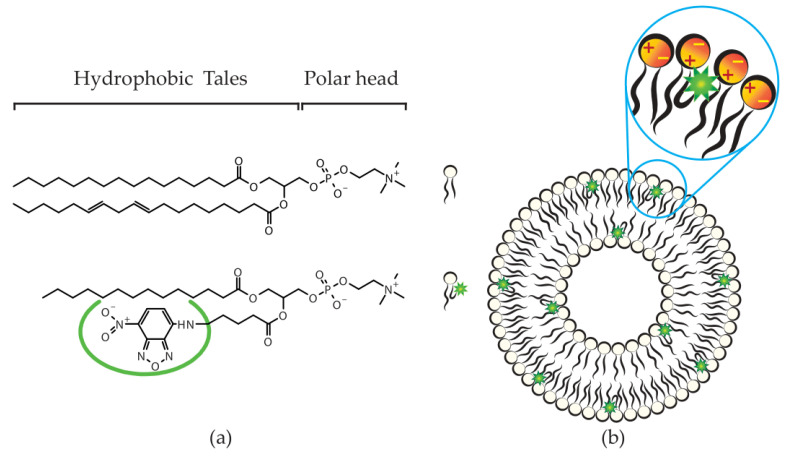
Liposomal formulation. Panel (**a**) (below) shows the fluorescently labeled phospholipid, for the visual comparison with the chemical structure of the main lipid ingredient in soy phosphatydilcholine (above). Panel (**b**) shows the expected random incorporation of the labeled lipid in the bilayer, according to minimal energy interaction and previous studies [34]. The molecules were drawn with ACD/ChemSketch (Freeware) 2019 2.1, according to the structures declared by the manufacturer.

In this study, we assessed the potential of quantitative phase microscopy as a suitable label-free technique for the characterization of nanocarriers. Liposomes were chosen as model carriers for their high therapeutic relevance [31] as well as their structural versatility. In conventional liposomes, such as those used in our study, phospholipids represent the structural repeated unit. Figure 1a (top) shows the chemical structure of a typical phospholipid, comprising a polar head (often a zwitterion) and hydrophobic tales (generally two carbon chains of various length). When hydrating phospholipids, their dual nature drives their self-assembly into vesicular structures with a hydrophobic bilayer enclosing a hydrophilic inner core (Figure 1b). Consequently, liposomes are often used both as solubilizers and as carriers, able to entrap and protect hydrophobic or/and hydrophilic active ingredients in their respective compartments. Their size, surface characteristics, and functionality can be tailored to address the challenges of the route of drug administration they are to be applied to [32].

From a technological point of view, liposomes are nanosized and almost transparent dynamic vesicles, very complex to image if not in a dried-out condition [33]. Furthermore, in quantitative phase imaging, their very nature causes only a slight delay in the light wavefront. This low signal becomes challenging to detect and interpret in laser-based QPM systems, thus a PTLS-equipped QPM setup was selected. To ensure that QPM images are trustworthy, we introduced a fluorescent marker within liposomal bilayers (Figure 1). The fluorescent signal emitted from the labeled liposomes was used to confirm the localization of liposomes on the interferogram. A fluorescent phospholipid (N) was selected as a marker due to its chemical structure similar to the natural lipid components within the liposomal bilayer (Figure 1a). Given its insolubility in water, the fluorescent lipid can only accommodate itself within the liposomal bilayer (Figure 1b).

## 2. Materials and Methods

### 2.1. Materials

1-myristoyl-2-{6-[(7-nitro-2-1,3-benzoxadiazol-4-yl)amino]hexanoyl}-sn-glycero-3-phosphocholine (14:0–06:0 NBD-PC, N) was purchased from Avanti Polar Lipids, Alabaster, AL, USA. Methanol, glucose, sucrose, and poly-L-lysine (PLL) were purchased from Sigma-Aldrich, Steinheim, Germany. Soy phosphatidylcholine (Lipoid S100, SPC) was obtained from Lipoid GmbH, Ludwigshafen, Germany.

### 2.2. Liposome Preparation

Liposomes were prepared following the film hydration method [15]. Low-pressure rotary evaporation of a methanol solution of SPC and fluorophore N (100:1) was performed using a Büchi rotary evaporator R-124 with vacuum pump V-700 (Büchi Labortechnik, Flawil, Switzerland). The thin film in the round-bottomed flask was then re-suspended by hand shaking in 2 M sucrose solution to the final concentration of SPC 10 mg/mL and N 0.1 mg/mL. Liposomal suspensions were then stored in the fridge at 4 °C. Prior to further processing, the size distribution was determined by combining the available techniques and settings.

### 2.3. Liposome Size Reduction

After overnight stabilization, the liposomes were processed by hand extrusion to tailor their size distribution [15]. Polycarbonate membranes (Nucleopore^®^) with sieving sizes of 800, 400, and 200 nm were used stepwise, as indicated in Table 1. Further overnight stabilization was ensured before the additional characterization steps.

### 2.4. Liposome Characterization: Size and ζ-Potential

Dynamic light scattering (DLS) was used to estimate size and zeta-potential distribution of the liposomal suspensions [35]. All dispersion were diluted 1:100 in 2 M glucose solution and analyzed with a Malvern Zetasizer Nano—ZS (Malvern, Oxford, UK).

An additional size characterization was performed on the unprocessed/filtered liposomes (N1), as the size distribution of the sample could not be reliably represented within the sensitivity range of the Malvern Zetasizer Nano—ZS (0.01–1 µm). A Particle Sizing System, Inc. Model 770 Accusizer (Santa Barbara, CA, USA), was used to estimate the size distribution in single-particle optical sensing. To optimize the sensitivity range of the instrument for the unknown particle size of the sample, both voltage thresholds were used, corresponding to size thresholds of 0.69 and 1.50 µm [36].

### 2.5. Liposome Immobilization for Imaging Purpose

**Figure A1:**
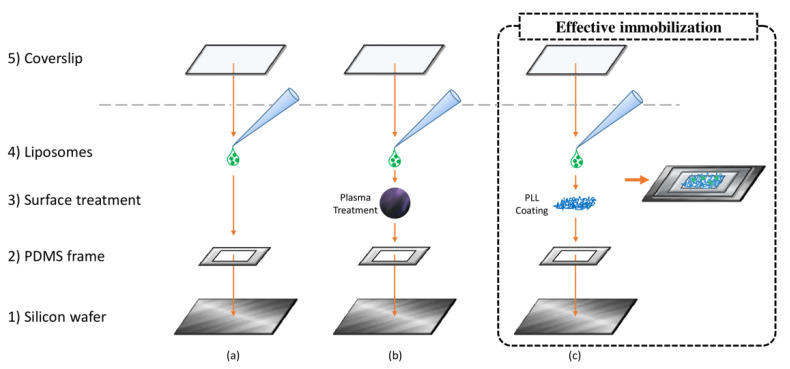
Non-dried immobilization setup for liposomes. In all cases, a silicon wafer was sterilized by heat and ethanol (**1**), and a PDMS frame was adjusted on top (**2**). The support was then used as such (**a**), after plasma treatment (**b**) or after coating with Poly-l-Lysine (**c**) (**3**). Few microliters of liposomal suspension were applied on top (**4**), and the system was allowed to equilibrate on the microscope stage. All setups were observed with and without coverslip sealing (**5**), but the presence of a coverslip in all cases allowed the suspension not to dry out.

Several immobilization strategies were attempted to obtain the liposomal suspension in monolayer without drying out the sample (Figure A1, in Appendix A). A silicon wafer with a PDMS frame was used as a support. Liposomes were diluted in a 2 M glucose solution to induce sedimentation, based on the difference in medium density inside and outside the bilayer [37]. Few microliters of liposomal suspension were applied inside the PDMS frame directly on the hydrophobic surface of the wafer, on top of a pre-jellified PLL coating, in a PLL suspension (co-jellification) and after plasma treatment of the wafer surface to increase its hydrophilicity. All setups were observed under the microscope, with and without coverslip sealing on top, and a long equilibration time was allowed for the system to stabilize the drifts on the microscope stage.

The best solution that was chosen for imaging and phase analysis was a combination of the previously used strategies. PLL was pipetted inside the PDMS frame and allowed to dry for 30 min. Few microliters of distilled water were used to rehydrate the PLL coating and then removed. The liposomal suspension pre-diluted in 2 M glucose to the final lipid concentration of 2 µg/mL was added on top of the coating. A coverslip was placed on top of the sample and sealed with nail polish. The wafer was then taped to the microscope stage and allowed to equilibrate for 30 min.

### 2.6. Imaging

**Figure 2:**
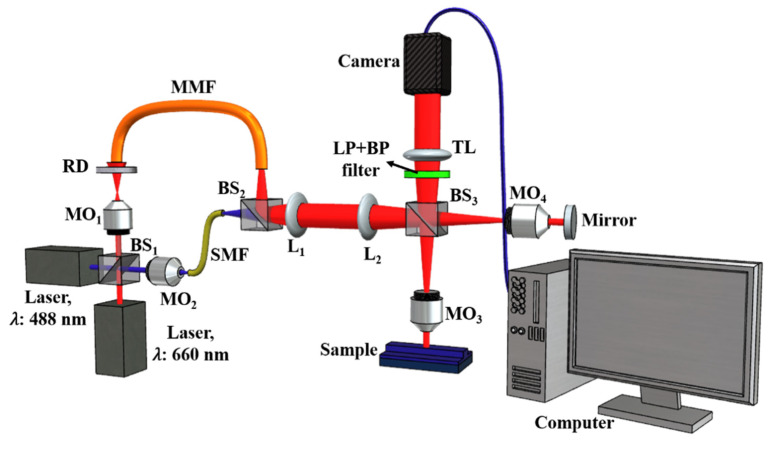
Schematic diagram of Linnik interferometer.

A schematic diagram of the imaging system used for QPM is shown in Figure 2. A nearly on-axis geometry of the microscope and a phase-shifting algorithm were chosen for high-resolution phase reconstruction of the nanosized liposomes. For fluorescence imaging, the liposomes were illuminated at 488 nm vacuum wavelength. The emitted fluorescent light alone was recorded by the CMOS camera with a combination of 488 nm long pass and (520/35) nm band pass filters. The 488 nm filter blocks the excitation light, and the bandpass filter allows only the emitted fluorescent light to reach the camera. QPM imaging was performed at 660 nm wavelength to exclude the possibility for the fluorescence label to affect the recovered phase maps, as previously shown [38].

With this technique, light from a laser source is passed through a rotating diffuser before coupling into a multi-mode fiber (MMF). To obtain a wide field of illumination at the sample plane S, the diverging beam from the MMF is collected using a combination of the lenses L_1_ and L_2_. The output from L_2_ is split into two halves using a beam splitter (BS). One half is focused at the back aperture of a microscope objective (MO_2_) to illuminate S. The reflected light off the sample plane is imaged onto a CMOS camera using BS and lens L_3_. This beam contains information about the sample under study and is referred to as the object beam. The second half known as reference beam is focused at the back aperture of the moving objective MO_3_ and is reflected off a reference mirror M. The reference beam is also imaged similarly onto the CMOS camera using BS and L_3_. The reference and object beams interfere in the CMOS camera to generate an interferogram.

The phase information about the sample under consideration is encoded in this interferogram and is retrieved using the phase-shifting algorithm method.

In this work, QPM was implemented in reflection mode, using a simple upright microscope. Therefore, samples were prepared on a reflecting substrate (wafer) and covered from the top with a cover glass. This configuration can be adapted in either inverted reflection mode or inverted transmission mode to accommodate different plates and dishes or even microfluidics devices (e.g., for cell imaging).

For the photobleaching and QPM experiment, utilizing the 1 µm-sized liposomes (N1), we acquired 26 fluorescence and phase datasets sequentially. The sample was exposed for approximately 10 s for each dataset, and photobleaching of liposomes took an average time of 4–5 min with a laser power of 20 mW on the sample plane. The acquisition time for one phase-shifted dataset using QPM was 1 s, and the switching time between fluorescence and phase imaging was around 30 s. Thus, the total time to acquire 26 fluorescence and phase datasets was approximately 18 min. 

### 2.7. Image Processing and Analysis

#### 2.7.1. Phase Retrieval Algorithm

The interferograms are 2D-modulated intensity (*I*) patterns. Mathematically, they can be defined as follows:

(1) $$I_{r}\left(x,y\right)=A_{r}\left(x,y\right)+B_{r}\left(x,y\right)cos\left[\phi \left(x,y\right)+\delta_{r}\right]$$

where the subscript *r* illustrates the *r*th phase-shifted interferogram (*r* = 1,2,3,…, N), $A_{r}\left(x,y\right)$ is the background, $B_{r}\left(x,y\right)$ is the modulation amplitude, ϕ(x,y) is the spatial phase information of the targeted specimen, and $\delta_{r}$ is the phase shift between the phase-shifted interferograms.

Assuming that $A_{r}\left(x,y\right)$ and $B_{r}\left(x,y\right)$ do not variate from one frame to the other, a new set of variables can be defined as:

$$a\left(x,y\right)=A_{r}\left(x,y\right),$$

$$b\left(x,y\right)=B_{r}\left(x,y\right)cos\phi \left(x,y\right),$$

$$c\left(x,y\right)=-B_{r}\left(x,y\right)sin\phi \left(x,y\right).$$

Equation (1) can thus be expressed as:

(2) $$I_{r}\left(x,y\right)=a\left(x,y\right)+b\left(x,y\right)cos\delta_{r}+c\left(x,y\right)sin\delta_{r}.$$

With $\delta_{r}$ known, the advanced iterative algorithm (AIA) [39] was used to solve the unknowns, and the spatial phase map of the specimen was recovered using the relation [39]:

(3) $$\phi \left(x,y\right)=tan^{-1}\left[\frac{-c\left(x,y\right)}{b\left(x,y\right)}\right].$$

The recovered phase map was then further utilized to calculate the thickness/height map of the sample, using the following expression:

(4) $$\phi \left(x,y\right)=\frac{2\pi }{\lambda }\left[n_{2}\left(x,y\right)-n_{1}\left(x,y\right)\right]h\left(x,y\right),$$

where λ is the wavelength of light used, $n_{2}\left(x,y\right)$ is the refractive index of the sample, $n_{1}\left(x,y\right)$ is the refractive index of the surrounding medium, and h(x,y) is the height/thickness of the sample. This equation implies that the phase retrieved from the interferogram is a product of the thickness of the sample and the refractive index difference between the sample and the surrounding medium.

#### 2.7.2. Size Distribution of Liposomes

A conventional bright field/dark field microscope cannot be used for the estimation of the size of nanosized objects due to their diffraction-limited image formation. The sizes of nanoobjects (<diffraction barrier) in the recorded images appear large and equal to the diffraction limit of the microscope. The limitation of a conventional microscope can be overcome indirectly by employing the highly sensitive QPM system, which has nanometric optical path length measurement sensitivity, for the estimation of the size of nanoobjects below the diffraction limit. Therefore, instead of directly measuring the XY size of the nanoobjects, one can measure their maximum phase/height values to estimate the size distribution by assuming their shape to be spherical. In order to estimate the size distribution of liposomes, the following steps are followed:

1. Recording of the phase-shifted interferograms of liposome samples.
2. High-resolution phase recovery by employing the AIA algorithm.
3. Removal of any background information from the recovered phase images either physically (through a reference/sample free interferogram) or numerically.
4. Convert the phase map into a height map by using Equation (4). The value of $\Delta n=\left(n_{2}-n_{1}\right)$ is assumed to be equal to 0.04.
5. Count the number of liposomes present in the recovered height map.
6. Find the maximum height values of all liposomes using the image-processing toolbox in MATLAB and utilize these values to draw a histogram plot.

## 3. Results

We present liposome characterization results using both conventional batch-mode techniques and QPM. We started with DLS characterization to obtain size distribution, zeta-potential, and PdI. We then assessed the QPM label-free characterization, consisting of imaging liposome localization, integrity, and shape, gaging the potential for single-particle-based size analysis.

### 3.1. Conventional Characterization of Labeled Liposomes

**Figure 3:**
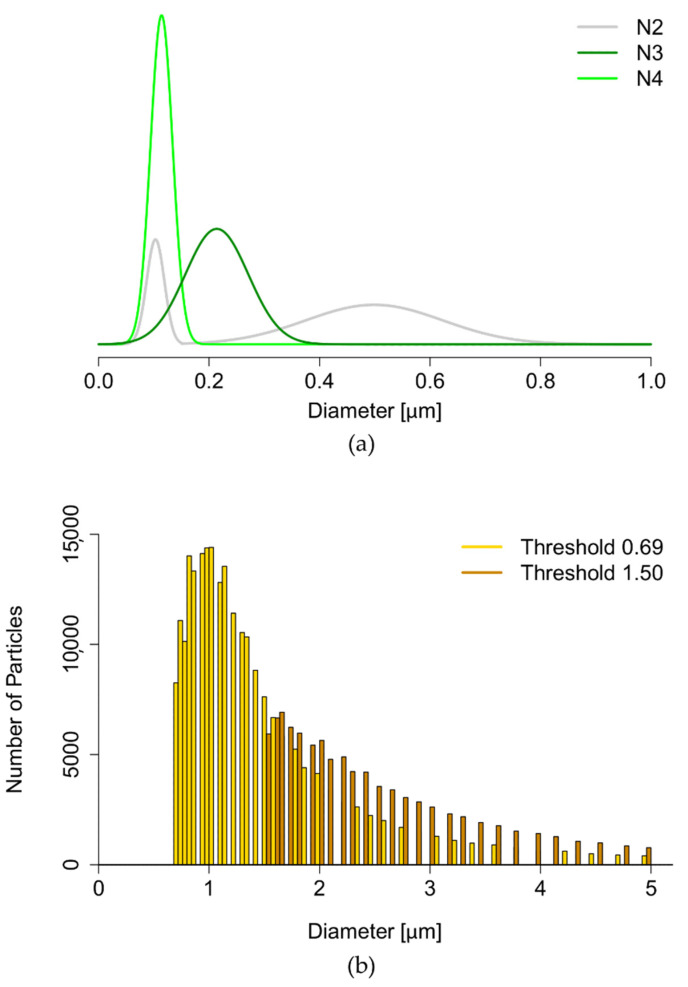
Conventional characterization of liposomes. (**a**) Intensity-weighted size distributions obtained with DLS (N2—gray, N3—dark green, N4—light green). (**b**) Number-weighted size distribution from single-particle optical sensing for N1, overlaying the result with size thresholds of 0.69 µm (gold) and 1.50 µm (brown).

From the original filtered batch (N1), three sequential size reduction steps were performed to obtain liposomes across the size spectra relevant for therapy (N2, N3, and N4). The corresponding size distributions are displayed in Figure 3. The upper panel shows the fitted intensity-weighted distributions to the different samples measured with DLS. As expected, the quality of the samples increased after longer processing, with sample N4 showing the best distribution (PdI = 0.11 ± 0.01), followed by N3 (PdI = 0.24 ± 0.02), while N2 showed a bimodal distribution, with PdI = 0.47 ± 0.04. No statistically acceptable distributions were obtained for N1 in the range 0.01–1 µm because of the high polydispersity of the sample (PdI = 0.85 ± 0.08), the interference of the bigger particles, and their tendency to sediment during the measurements [40]. For this reason, N1 was measured with single-particle optical sensing, a complimentary conventional characterization with a size sensitivity range shifted towards micrometer-sized particles. This is represented in the lower panel of Figure 3 as a number-weighted distribution, with the two available voltage thresholds showcasing truncated curves, with mode of 1 µm. Interestingly, after nanosizing the vesicles, the size results did not match the expected values. Table 2 shows the expected ranges of size, PdI, and zeta-potential based on the literature [15,35,41] for corresponding extrusions of non-labeled liposomes. In particular, the intermediate processing (N2: 4 × 800 extrusion) did not result in a stable formulation. Furthermore, the zeta-potential exhibited strongly negative values compared to the neutral values reported in the literature for the liposomes extruded in a similar manner. The increased zeta potential values in our liposomes (N1–N4) might be contributed by the surface-available fluorescent moiety [34]. Table 2 contains an overview of the characterization (size interval, PdI, and ζ-potential), together with previously published values for non-labeled liposomes, for comparison.

### 3.2. QPM Label-Free Characterization of Liposomes

To complement the conventional characterization, liposomes were successfully immobilized on PLL-coated silicon wafers and imaged in fluorescence and phase modes for a direct comparison of the viability of the label-free technique.

**Figure 4:**
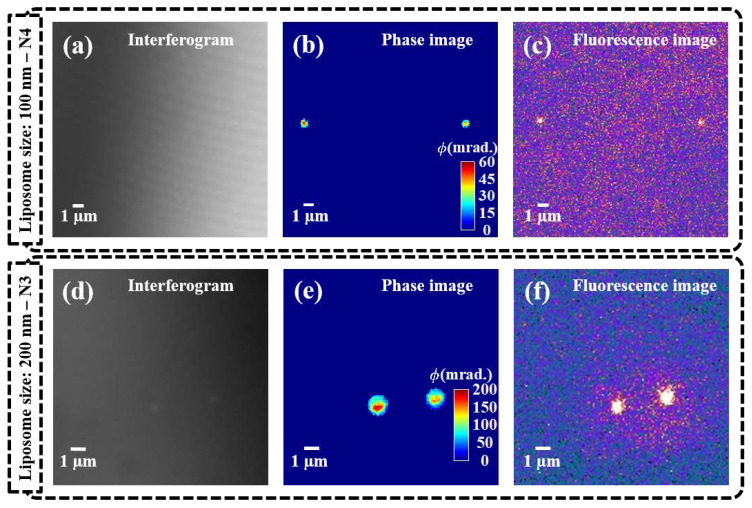
Single-liposome imaging. Two representative liposomes are shown in both phase and fluorescence imaging. The upper panels show the 100 nm liposomes (N4), while the lower panels display the 200 nm liposomes (N3). From left to right: (**a**,**d**) show the interferograms recorded in QPM; (**b**,**e**) the phase images retrieved from the interferograms (with calibration bar in milliradians); (**c**,**f**) the fluorescence images.

The localization of liposomes using QPM is displayed in Figure 4, where phase imaging is opposed to fluorescence imaging for two different liposome sizes—100 and 200 nm. The interferograms (Figure 4a,d) and retrieved phase maps (Figure 4b,e) show it is possible to distinguish the different sizes of liposomes below the diffraction limit of light. The calibration bars for the phase images show a phase max of 60 and 200 mrad for the samples N4 and N3, respectively. This translates to diameter values of 74 and 212 nm, once fixed to 0.04 the refractive index difference between the liposomes and the medium (Δ*n*).

**Figure 5:**
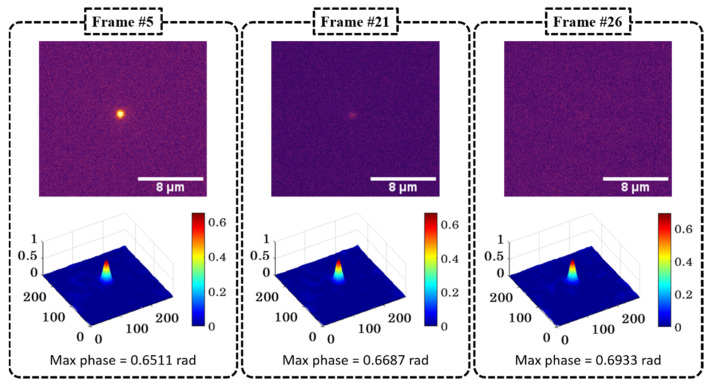
Single-liposome characterization over time. The sample was illuminated beyond photobleaching of the fluorescent signal without recording changes in the phase interferogram. The fluorescence images (**top**) and the phase reconstructions (**bottom**) of three sample frames (#5, #21 and #26) are presented.

To assess the performance of QPM vs. fluorescence for prolonged imaging, the same liposome (from N1) was followed with both modes, as shown in Figure 5. The upper panels show photobleaching over time with complete signal loss and consequent loss of tracking of the liposome localization by frame 26. The lower panels display the phase maps, which continue to show the presence of a liposome even after photobleaching. No relevant structural deformations were detected throughout the process, suggesting that the loss of fluorescence did not affect the integrity of the liposome. The slight variation in the maximum phase values of the liposome as a function of time could be due to minute defocusing while acquiring the sequence of fluorescence and phase data.

**Figure 6:**
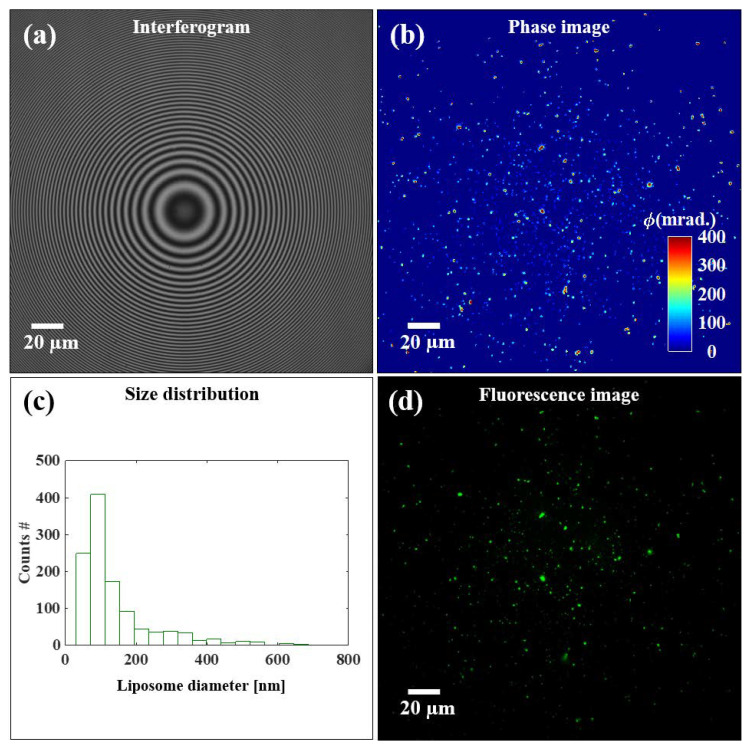
Full field of view of the liposome sample (N3). Panel (**a**) recorded interferogram; (**b**) phase image; (**c**) size distribution obtained from the sample phase image shown, and (**d**) fluorescence image.

When looking at the full field of view in Figure 6, we can better see how phase imaging allows for a more accurate localization of liposomes, independently of the fluorescent signal. In fact, the phase signal was present also for those liposomes that carried too little or no fluorescent label, allowing for a more accurate estimation of size distribution. The details of image processing for the estimation of size distribution are given in Section 2.7.2.

## 4. Discussion

Lipid-based nanoparticles such as liposomes are widely used as nanomedicines because of their high biosafety. The use of lipids naturally present in cells and the adjustable size of the final particles make them relevant for both topical and systemic drug delivery. Furthermore, the presence of both a lipid bilayer and a water-based core solution allows for the loading of both hydrophobic and/or hydrophilic drugs, with great potential in many therapeutic challenges [32,42]. However, the very same versatility that contributes to their therapeutic relevance can hamper the technological characterization necessary for development processes, prior to biological testing [7]. In this work, we investigated some of the challenges related to conventional characterization methods (DLS and fluorescence-based assays) such as polydispersed samples, out-of-range particles, and labeling-dependent behavior. Furthermore, we propose QPM as a complementary technique for a deeper characterization of a nanosystem, based on a label-free single-particle analysis. Since no literature data are available on the use of QPM for liposomes characterization, at this stage, we included a fluorescent lipid (Figure 1b) within the liposomal bilayers to assist in liposomal localization during QPM characterization.

The characterization of unprocessed liposomes (N1) highlights the major challenges of conventional batch-mode analyses. Size, surface charge, and polydispersity of liposomal formulations are conventionally determined by harnessing their fast Brownian movement through intensity detection of backscattered light (DLS) [40]. Common lab-bench instruments for this purpose (e.g., Malvern Zetasizer Nano—ZS, used in this work) have a sensitivity range in the nanoscale, up to 1 µm, and their built-in Cumulants algorithm uses Gaussian fitting for the estimation of the size distribution, with resolution limited by a factor of 3 [7]. Because we used the thin-film hydration method to prepare the liposomal formulations, the re-suspension of the lipid film in the water phase was expected to form multilamellar/multivesicular macroparticles with great variability in size [43]. Hence, in the N1 sample, (I) the presence of big vesicular bodies (>1 µm) was interpreted by the software as dust contamination and excluded from the reading. (II) The tendency of these big particles to sediment during the measurement itself was translated into z-average trending by 10–30% over technical replicates of the same measurement. (III) The high polydispersity (estimated as PdI = 0.85 ± 0.08) prevented a statistically acceptable fitting, resulting in a poor quality of the measurement.

For a better characterization of N1, we resorted to single-particle optical sensing, using both the available voltage thresholds to increase the accuracy of the size determination over the whole range of 0.69 to 5 µm (according to previously optimized protocols [36]). The resulting size distribution (Figure 3b) showed a truncated number-weighted distribution that still brings challenges for its interpretation. In fact, (I) the truncated distribution showed clear missing information below the lower sensitivity threshold, and (II) this number-weighted distribution was hard to compare to the DLS intensity-weighted distributions obtained for the other samples of the experiment (N2, N3, and N4, Figure 3a) [40].

Combining all available information from conventional characterization (Table 2), we noticed an unexpected size outcome for each processing (Table 1). The overall measured values of size were found to be smaller than expected from the unprocessed batch N1, down to N3 and N4—sizes that are normally very difficult to achieve with hand extrusion or, at least, require longer processing [44]. Both the smaller sizes and the instability of the batches with intermediate processing (N2) can be explained by the presence of the fluorophore in the bilayer, as this adds a layer of complexity to nanoparticle characterization. Although the use of fluorescent probes has great potential to track nanoparticle behavior in a biological environment, it comes with technological challenges in handling the formulation, such as (I) interference in DLS measurements [40], (II) surface modifications [16], (III) thermal instability [15], (IV) possible fluorophore detachment [11,17], and ultimately, (V) loss of fluorescence specificity [45].

For validation purposes, a fluorescent phospholipid (N) was chosen to ensure the least invasive labeling strategy for the phospholipid bilayer of liposomes. However, although chemically linked to the hydrophobic chain of the phospholipid (Figure 1a), the NBD fluorescent moiety was shown to backflip towards the polar heads of the bilayer (Figure 1b) [34]. The increased efficiency of size reduction processing, such as hand extrusion, is therefore due to the behavior of the fluorescent moiety, which affects fluidity and viscosity of the bilayer [46]. At the same time, the position of the fluorescent moiety has a high chance of interfering with the position of the zwitterionic charges on the surface of the bilayer (Figure 1b—zoom in), consequently affecting the electrostatic interactions between the bilayer and the isotonic complex medium, thus explaining the relevant negativity of the surface [47].

To overcome the fluorophore-related downsides in nanomedicine, such as the abovementioned technological challenges, the risk of photobleaching, and the potential photo-toxicity, we focused on assessing the potential of QPM as a label-free characterization technique. As we aimed to image small liposomes (close to and below the resolution limit of light, for N3 and N4 respectively), we chose high spatial resolution over temporal resolution with on-axis microscope and phase-shifting algorithm for high-resolution and highly sensitive phase reconstruction from the recorded interferogram [22,48,49]. We achieved a successful immobilization of liposomes by pre-coating the silicon wafer support with Poly-L-Lysine. This trick allowed for non-dried-out imaging conditions, which are known to significantly affect the properties and shapes of liposomes [33]. Based on the effective immobilization of liposomes and the high spatial resolution of the setup, both diffraction-limited samples could be localized in the phase map, and their sizes differentiated (between N3 and N4) (Figure 4). As the fluorescence images in Figure 4 show, smaller liposomes presented a smaller load of dye, increasing the risk of losing track of them when relying on the sole fluorescence-based tracing in biological environment. Figure 5 shows that the rapid photobleaching of the fluorescence dye over time did not cause changes in the shape and structural integrity of the liposomes. Hence, not only is QPM independent of a fluorescent label for the detection of liposomes, but also it shows superior tracking abilities over time, as the loss of fluorescence signal does not translate in the absence/degradation of the original liposome. Furthermore, Figure 6 shows a full field of view of immobilized liposomes, both in fluorescence and in phase imaging. The higher number of liposomes visible in the phase map confirms the higher accuracy of detection that cannot be expected in label-dependent detection. Indeed, when adding both labeled and non-labeled lipids in the initial mixture, prior to evaporation and rehydration, a random distribution of the fluorescent moiety is to be expected within the sample (Figure 1b). However, the processing by hand extrusion involves “peeling” and rearrangements of the membranes that will “dilute” the dye over a larger number of smaller liposomes, potentially preventing the detection of some of them [50].

**Figure A2:**
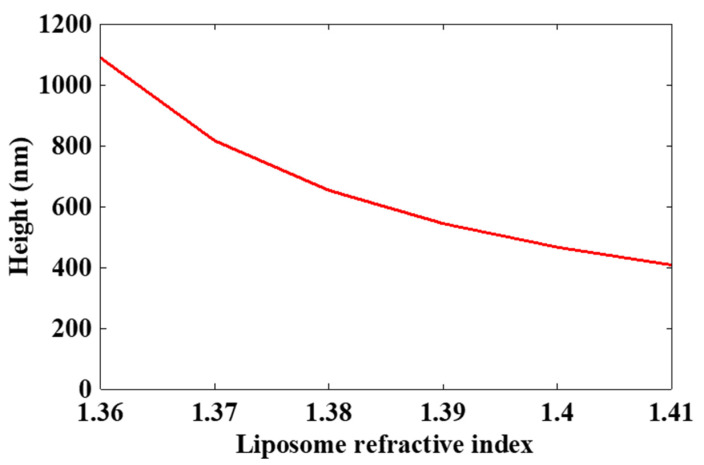
Variation of liposome diameter (height) as a function of the refractive index.

From the phase image, it is possible to obtain a size estimation of liposomes based on single-particle analysis. Choosing a 0.4 Δ*n* between medium and liposomes, we obtained a distribution centered around 100 nm for the N3 sample. The lower size estimation when comparing to DLS can be explained by different factors. Firstly, we compared a number-weighted (QPM) with an intensity-weighted (DLS) distribution. In the latter case, as the intensity is proportional to the power of 6 of the liposome diameter (d^6^), bigger particles will contribute much more to the intensity, resulting in an upwards bias, as previously shown when comparing DLS with TEM results [51]. Secondly, choosing an improper value for the refractive index of both medium and liposomes can lead to biased size estimates. This is a challenging aspect for the characterization of liposomes, as they are non-solid particles made of lipid mixtures. Figure A2 in Appendix A shows the variation of the diameter with the liposome refractive index, with downward bias as the refractive index increases. Finally, it has been shown that sub-diffraction structures can be associated with size underestimation due to the possible loss of high-frequency information during image detection [52].

Even though some optimization steps may still be required to fully utilize QPM, we have shown the potential of the method in complementing the conventional characterization of nanocarriers. The non-dried setup here used for the immobilization of liposomes can potentially be applied for the characterization of different types of liposomes, as well as other types of lipid-based vesicles. We would expect this methodology to provide a deeper insight into the characteristics of the vesicles in their hydrated stated with rather intact morphology—as opposed to the conventional dried TEM samples. Furthermore, knowing the size of the nanosystem (thickness h(x,y) in Equation (4)), QPM interferograms could be used to retrieve variations in the refractive index, thus expanding the possible applications of this technique for the morphological analysis of nanoparticles. Most interesting examples in lipid-based nanomedicine could be (I) vesicles bearing edge activators, such as deformable liposomes [35], (II) vesicles comprising glycerol within the bilayers, i.e., glycerosomes [53], (III) polymer-immobilized vesicles, such as hyalurosomes [54], (IV) surface-modified vesicles, such as liposomes for targeted immunotherapy [32], and more. However, at this stage, we can only speculate whether QPM would be easily applicable in the characterization of lipid-based vesicles where the lipid bilayers are more complex than in our case.

Future perspectives include addressing the size underestimation for sub-diffraction particles and optimizing the trade-off between spatial and temporal resolution to follow the behavior of moving nanoparticles in biological environments. This would not only allow improvement in the pre-biological characterization of nanomedicine but also provide the missing link between the technological characterization we reported here and the analysis of cellular morphology after nanoparticles treatment, recently reported to be feasible utilizing QPM [21,55,56]. Thus, QPM shows a great potential for all-in-one label-free characterization of properties and behavior of drug delivery systems.

## 5. Conclusions

The versatility of liposomal formulations makes their characterization challenging at times. Robust and easy-to-perform conventional techniques can fail to provide accurate results in case of high polydispersity or out-of-range nanoparticles. The characterization of nanomedicines’ behavior in a biological environment—often based on the fluorescent marker incorporated within the nanocarrier—bears the risks of losing tracing specificity, causing photobleaching, and imparting photo-toxicity to the sample. QPM is hereby introduced as a complementary characterization technique with the potential of localizing, tracking over time, and allowing further image processing to obtain size distributions based on single-particle analyses.

## Figures and Tables

**Table 1:** Liposome processing to size reduction.

Formulation	Extrusion
N1	1 × 800 nm ^1^
N2	4 × 800 nm
N3	4 × 800 nm, 4 × 400 nm
N4	4 × 800 nm, 4 × 400 nm, 4 × 200 nm

^1^ Single filtration to exclude potential particle contaminants on the manufacturing.

**Table 2:** Conventional characterization of liposomes (Lip). Measured values (left) refer to the N-labeled formulations analyzed in this work. Expected values (right) show ranges commonly reported in the literature [15,35,41] for the correspondent processing of non-labeled liposomes. Values are expressed as mean ± standard deviation, unless otherwise indicated.

Lip	Measured Values			Expected Values		
	Size [nm]	PdI	ζ-Potential [mV]	Size [nm]	PdI	ζ-Potential [mV]
N1	1040 ^1^	0.85 ± 0.08	−55.7 ± 6.3	>>1000	1	[−5, +5]
N2	499 ± 124 (74.5%) ^2^ 103 ± 16 (25.5%)	0.47 ± 0.04	−59.8 ± 5.1	600–800	<0.250	[−5, +5]
N3	214 ± 57	0.24 ± 0.02	−57.1 ± 6.7	300–500	<0.250	[−5, +5]
N4	114 ± 20	0.11 ± 0.01	−55.4 ± 6.6	150–350	<0.250	[−5, +5]

^1^ Mode (peak) of the truncated distribution (number-weighted), Figure 3b. ^2^ Bimodal distribution described with intensity percentage for each peak in brackets.

## Data Availability

Not applicable.
